# Correction to ‘The contractile patterns, anatomy and physiology of the hyoid musculature change longitudinally through infancy’ (2022) by Mayerl *et al.*

**DOI:** 10.1098/rspb.2022.1936

**Published:** 2022-10-12

**Authors:** C. J. Mayerl, K. E. Steer, A. M. Chava, L. E. Bond, C. E. Edmonds, F. D. H. Gould, B. M. Stricklen, T. L. Hieronymus, R. Z. German

*Proc. R. Soc. B* **288**, 20210052. (Published online 10 March 2021) (https://doi.org/10.1098/rspb.2021.0052)

There is a typo in the last name of one of the authors:

T. L. Hieronymous should read T. L. Hieronymus

